# Stabilization of the gp120 V3 loop through hydrophobic interactions reduces the immunodominant V3-directed non-neutralizing response to HIV-1 envelope trimers

**DOI:** 10.1074/jbc.RA117.000709

**Published:** 2017-12-07

**Authors:** Steven W. de Taeye, Alba Torrents de la Peña, Andrea Vecchione, Enzo Scutigliani, Kwinten Sliepen, Judith A. Burger, Patricia van der Woude, Anna Schorcht, Edith E. Schermer, Marit J. van Gils, Celia C. LaBranche, David C. Montefiori, Ian A. Wilson, John P. Moore, Andrew B. Ward, Rogier W. Sanders

**Affiliations:** From the ‡ Department of Medical Microbiology, Academic Medical Center, University of Amsterdam, Amsterdam, 1105 AZ, The Netherlands,; the § Department of Surgery, Duke University Medical Center, Durham, North Carolina 27710,; the ¶ Department of Integrative Structural and Computational Biology, Scripps CHAVI-ID, IAVI Neutralizing Antibody Center and Collaboration for AIDS Vaccine Discovery, Scripps Research Institute, La Jolla, California 92037, and; the ‖ Department of Microbiology and Immunology, Weill Medical College of Cornell University, New York, New York 10021

**Keywords:** human immunodeficiency virus (HIV), vaccine development, HIV envelope glycoprotein trimer, protein design, glycoprotein structure, structure-function, V3 domain

## Abstract

To provide protective immunity against circulating primary HIV-1 strains, a vaccine most likely has to induce broadly neutralizing antibodies to the HIV-1 envelope glycoprotein (Env) spike. Recombinant Env trimers such as the prototype BG505 SOSIP.664 that closely mimic the native Env spike can induce autologous neutralizing antibodies (NAbs) against relatively resistant (tier 2) primary viruses. Ideally, Env immunogens should present broadly neutralizing antibody epitopes but limit the presentation of immunodominant non-NAb epitopes that might induce off-target and potentially interfering responses. The V3 loop in gp120 is such a non-NAb epitope that can effectively elicit non-NAbs when animals are immunized with SOSIP.664 trimers. V3 immunogenicity can be diminished, but not abolished, by reducing the conformational flexibility of trimers via targeted sequence changes, including an A316W substitution in V3, that create the SOSIP.v4.1 and SOSIP.v5.2 variants. Here, we further modified these trimer designs by introducing leucine residues at V3 positions 306 and 308 to create hydrophobic interactions with the tryptophan residue at position 316 and with other topologically proximal sites in the V1V2 domain. Together, these modifications further stabilized the resulting SOSIP.v5.2 S306L/R308L trimers in the prefusion state in which V3 is sequestered. When we tested these trimers as immunogens in rabbits, the induction of V3 non-NAbs was significantly reduced compared with the SOSIP.v5.2 trimers and even more so compared with the SOSIP.664 prototype, without affecting the autologous NAb response. Hence, these additional trimer sequence modifications may be beneficial for immunization strategies that seek to minimize off-target non-NAb responses.

## Introduction

A vaccine that provides protective immunity against the majority of diverse circulating strains would probably be the most effective way to halt the HIV-1 pandemic (1). Neutralizing antibodies (NAbs)^4^ are the correlate of protection for most licensed vaccines and are a main focus of HIV-1 vaccine research (2). The identification of multiple broadly neutralizing antibodies (bNAbs) from infected individuals, including ones that can counter up to 90% of circulating viruses, underpins the goal of designing envelope glycoprotein (Env)-based immunogens intended to elicit a similar response (2–5).

The Env trimer has acquired several effective mechanisms for evading humoral immunity by hindering the induction of NAbs, limiting the binding of any that are induced, and facilitating escape from any that do bind. For example, the trimer's conserved protein domains are masked by a dense glycan shield and by flexible and sequence variable loops (6–8). Furthermore, during HIV-1 infection, non-functional, misfolded, degraded, or under-processed forms of Env may act as decoys to the humoral immune system and result in the production of Abs against irrelevant targets. Thus, these non-native Env forms present epitopes that are not accessible or formed on the functional Env trimer (9–17). As a consequence of the defense mechanisms described above, most Abs raised during the early phase of HIV-1 infection are directed against neutralization-irrelevant epitopes on gp41 and the V3 region of gp120 (3). Thus, V3 is considered to be an immunodominant component of Env (3, 18, 19). Although V3 Abs efficiently neutralize a subset of relatively sensitive (termed tier 1) isolates, they are non-neutralizing for most of the more neutralization-resistant (tier 2) primary viruses that are the vaccine targets (20). Accordingly, V3 Abs do not generally drive the selection of escape mutants in infected people (21, 22).

Although we now know a great deal about the properties of bNAbs and their epitopes, we do not yet know how to induce them by immunization (23, 24). One approach to this substantive problem is based on the assumption that the Env immunogen should present bNAb epitopes efficiently. The SOSIP design of soluble, recombinant native-like trimers has this property, exemplified by the BG505 SOSIP.664 construct that has now been extensively studied structurally and as an immunogen (13, 14, 25). These trimers, and multiple others of various genotypes, display almost all known bNAb epitopes except those in the MPER, which is a very hydrophobic region at the C terminus of the ectodomain that is not included in the design. When tested as immunogens in rabbits, guinea pigs, and macaques, SOSIP.664 trimers of various genotypes induce autologous tier 2 NAbs at variable but often quite high titers, but they only elicit heterologous tier 2 responses sporadically (14, 25, 26). In contrast, BG505 SOSIP.664 trimers do induce bNAbs in cows, probably related to the presence of the unusual ultralong CDRH3 domains in the bovine Ab repertoire (27).

The SOSIP.664 trimers also induce non-NAb responses, dominated by Abs against V3 that cross-neutralize tier 1 viruses but not their more resistant and more relevant tier 2 and above counterparts (14, 25). The V3 region is a highly flexible region on the Env trimer and is part of the co-receptor binding site. In the prefusion state of the native trimer, the V3 region is tucked away in a hydrophobic pocket underneath the variable domains 1 and 2 (V1 and V2) at the trimer apex (28–30). The spontaneous sampling of more open conformations in Env trimers is thought to expose the immunodominant V3 region, resulting in a strong V3-directed Ab response in animals (13, 31, 32). We and others have found various strategies to reduce exposure of the V3 domain on the Env trimer and reduce the exposure of V3 non-NAb epitopes. In one study Env trimers were stabilized in complex with a quaternary dependent V2-apex Ab, PGT145, resulting in significantly lower elicitation of V3-directed tier 1A Abs when immunized in guinea pigs (33). More recently, Ringe *et al.* (34) showed that the introduction of two glycans in the V3 loop also reduced the induction of V3-directed non-NAbs in rabbits. Many other studies sought to reduce the exposure of the immunogenic V3 loop by stabilizing the closed prefusion conformation of the Env trimer (25, 26, 33, 35–40). We previously identified a stabilizing mutation within the V3 region, A316W, that increases hydrophobic packing of the V3 loop, effectively sequestering the V3 loop in its prefusion conformation in the hydrophobic pocket underneath the V1V2 domains (25). This A316W substitution is included in the next-generation SOSIP.v4 design that we now routinely apply to new Env trimers. When comparing clade A BG505 SOSIP.v4 and clade B AMC008 and B41 SOSIP.v4 trimers with their parental SOSIP.664 trimers, the stabilized SOSIP.v4 immunogens elicited similar levels of autologous NAbs, but substantially reduced levels of V3-dominated tier 1A NAbs in rabbits, illustrating that we indeed achieved a reduction in V3 immunodominance (25). However, the effect was not absolute, and V3 specificities remained abundant. Furthermore, the effect was less pronounced in macaques (41).

We hypothesized that HIV-1 vaccine programs aimed at inducing bNAbs by using SOSIP trimers could be assisted by further limiting V3-directed non-NAb responses. To further increase hydrophobic packing of the V3 region, thereby reducing V3 immunogenicity, we identified additional hydrophobic mutations in the V3 region via structure-based design. Two hydrophobic mutations in the V3 loop of BG505 SOSIP trimers, S306L and R308L, that increased the stability of BG505 SOSIP immunogens, further reduced binding of V3 non-NAbs to the trimers and severely impaired V3-directed, tier 1A NAb responses in vaccinated rabbits. These new V3 stabilized HIV-1 Env immunogens are useful as platforms for immunogen design aimed at inducing bNAbs.

## Results

### Hydrophobic substitutions in V3 further stabilize this region of the trimer

**Figure 1.**
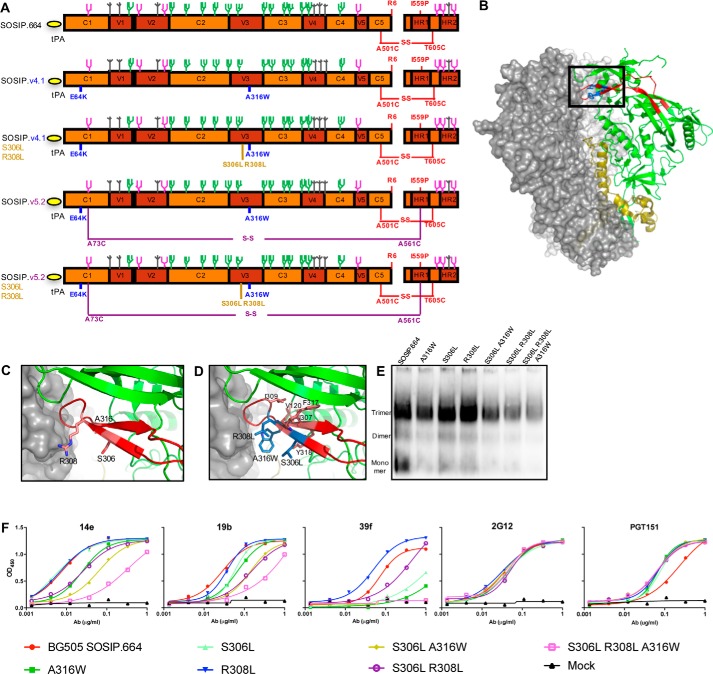
**Design and effect of hydrophobic substitutions in the V3 domain.** *A*, linear representation of V3-stabilized BG505 SOSIP variants. The SOSIP.664 mutations are indicated in *red*, the SOSIP.v4.1 mutations (E64K and A316W) are in *blue*, the SOSIP.v5.2 mutations (A73C–A561C) are in *purple*, and the new hydrophobic V3 mutations are in *light brown* (13, 25, 42). The assignment of glycans was based on Behrens *et al.* (6) and discriminates high-mannose glycans (*green*), processed complex and hybrid glycans (*magenta*), and glycans of unknown composition (*gray*). *B*, side view of the crystal structure of BG505 SOSIP.664 (93). Two protomers are shown in surface rendering, one in *white* and one in *gray*, whereas the third protomer is represented in *ribbon*. In this third protomer various subdomains are colored as follows: gp41 in *dark yellow*, gp120 in *green*, and the V3 region in *red*. The hydrophobic V3 mutations, S306L, R308L, and A316W, were introduced into the crystal structure by *in silico* mutagenesis using PyMOL and are depicted in *blue. C* and *D*, details of the V3 region of the BG505 SOSIP.664 trimer, derived from the crystal structure. In *C*, residues Ser^306^, Arg^308^, and Ala^316^ are shown as *sticks*, whereas *D* shows the impact of introducing the S306L, R308L, and A316W substitutions (shown in *blue*) by *in silico* mutagenesis. *E*, BN-PAGE analysis of unpurified BG505 SOSIP.664-D7324 V3 mutants, followed by Western blotting with bNAb 2G12. *F*, D7324-capture ELISA analysis of Ab reactivity with the same trimer variants.

We previously found that the introduction of an A316W mutation into V3 reduced the exposure of this Env domain on the trimer surface and thereby reduced the induction of V3-directed non-NAbs by ∼10-fold when the resulting SOSIP.v4 trimers were tested as immunogens in mice and rabbits, but to a lesser extent in macaques (25, 41). To further decrease V3 immunogenicity, we carried out a structure-guided redesign in the V3 region of the BG505 SOSIP.v4.1 and SOSIP.v5.2 trimers that already contain the A316W substitution (Fig. 1*A*) (25, 42). In the prefusion state of the trimer, the V3 region is buried within a pocket formed by the V1V2 loop and stem of the same protomer and the V1V2 loop from a second protomer (28, 30, 43, 44). More specifically, the V3 crown predominantly participates in intraprotomer hydrophobic interactions with the V1V2 loop and with the N-terminal region of the V1V2 stem of the same protomer (44). Disrupting these cross-domain hydrophobic interactions changes the packing of the V3 region, destabilizes its location in the pocket, and triggers a transition toward alternative, energetically favorable conformations in which V3 is now surface-exposed (44).

To strengthen the hydrophobic interactions that maintain the burial of the V3 region in the pocket formed by the adjacent V1V2 elements, we introduced leucine residues at V3 positions 306 and 308 (Fig. 1, *A–D*). In the original BG505 V3 sequence, serine 306 and arginine 308 are located on the N-terminal flank of the V3 crown and face the inner domain of gp120 in a manner similar to how residue 316 (originally alanine, now changed to tryptophan) is oriented on the other flank of the V3 crown (Fig. 1*C*). Our selection of the leucine substitutions was governed by the outcome of modeling different hydrophobic residues at positions 306 and 308 *in silico*. Thus, leucine residues introduced a substantial amount of hydrophobic mass without clashing with neighboring amino acids; in contrast, introducing residues with bulky, aromatic side chains did create clashes (Fig. 1*D*). The *in silico* models showed that all three of the introduced hydrophobic residues, Leu^306^, Leu^308^, and Trp^316^, are in close contact (<5 Å) with each other and with other hydrophobic residues (Val^120^, Ile^307^, Ile^309^, Phe^317^, and Tyr^318^). The models therefore support our hypothesis that these mutations should strengthen the hydrophobic interactions of the V3 domain and stabilize the V3 structure (Fig. 1*D* and Table 1).

**Table 1:** **BG505 SOSIP.664 trimer residues in contact with positions 306, 308, and 316** We used PyMOL to identify the residues in contact with (distance of <4 or <5 Å) amino acids Ser^306^, Arg^308^, and Ala^316^ in the structure of the wild-type BG505 SOSIP.664 trimer (Protein Data Bank code 5CEZ) (93) and then employed an *in silico* model to assess the contact residues for the hydrophobic substitutions at these positions, i.e., Leu^306^, Leu^308^, and Trp^316^ (94). Residues 306, 308, and 316 are highlighted in bold type.

Residue	Contact residues	
	<4 Å	<5 Å
Ser^306^	Lys^305^, Ile^307^, Phe^317^	Arg^304^, Lys^305^, Ile^307^, Arg^308^, Phe^317^, Tyr^318^, Ala^319^
S306L	Lys^305^, Ile^307^, Phe^317^	Arg^304^, Lys^305^, Ile^307^, **Leu^308^**, **Trp^316^**, Phe^317^, Tyr^318^, Ala^319^
Arg^308^	Thr^162^, Glu^164^, Gln^170^, Ile^307^, Ile^309^, Gly^312^, Gln^315^	Thr^162^, Thr^163^, Glu^164^, Gln^170^, Ser^306^, Ile^307^, Ile^309^, Gly^312^, Gln^315^, Ala^316^, Phe^317^, (Asn^197^, adjacent protomer)
R308L	Thr^162^, Glu^164^, Gln^170^, Ile^307^, Ile^309^, Gly^312^, Gln^315^, **Trp^316^**	Thr^162^, Thr^163^, Glu^164^, Gln^170^, **Leu^306^**, Ile^307^, Ile^309^, Gly^312^, Gln^315^, **Trp^316^**, Phe^317^ (Asn^197^, adjacent protomer)
Ala^316^	Gln^203^, Ile^307^, Gly^314^, Gln^315^, Phe^317^, Tyr^318^	Val^120^, Gln^203^, Ile^307^, Arg^308^, Ile^309^, Gly^314^, Gln^315^, Phe^317^, Tyr^318^
A316W	Gln^203^, Ile^307^, **Leu^308^**, Gly^314^, Gln^315^, Phe^317^, Tyr^318^	Val^120^, Gln^203^, **Leu^306^**, Ile^307^, **Leu^308^**, Ile^309^, Gly^312^, Gly^314^, Gln^315^, Phe^317^, Tyr^318^

We first introduced the S306L, R308L, and A316W substitutions into a D7324-tagged version of the BG505 SOSIP.664 trimer, alone and in combination; transiently expressed the proteins in 293T cells; and analyzed the unpurified Env proteins in the culture supernatants. Assessed by BN-PAGE gel electrophoresis and Western blotting, each of the single mutants formed trimers efficiently, but the two double mutants S306L/A316W and S306L/R308L and the S306L/R308L/A316W triple mutant were somewhat less efficiently expressed compared with the unmodified SOSIP.664 trimer (Fig. 1*E*).

We also used the unpurified Env proteins to gauge how the mutations affected the antigenicity of V3 epitopes, as judged by the reactivity of three V3-directed non-NAbs (19b, 14e, and 39f) in a D7324-capture ELISA. In this type of assay, the V3 region of SOSIP.664 trimers is highly accessible (13). The individual S306L and R308L point substitutions did not markedly affect the trimer binding of the V3 non-NAbs (Fig. 1*F*). However, when introduced together (S306L/R308L), and more so in combination with the A316W change (S306L/R308L/A316W), they greatly reduced or completely abrogated the binding of all three V3 non-NAbs without affecting the reactivity of the 2G12 bNAb with its oligomannose patch epitope and the quaternary dependent bNAb PGT151 (Fig. 1*F*). These data suggest that hydrophobic residues at positions 306, 308, and 316 help maintain a sequestered V3 conformation in which non-NAb epitopes are inaccessible on the trimer surface. The alternative explanation that the non-NAb epitopes are directly affected by the substitutions is addressed below by studies using purified trimers.

### Introduction of hydrophobic residues in V3 reduces Env function

**Figure 2.**
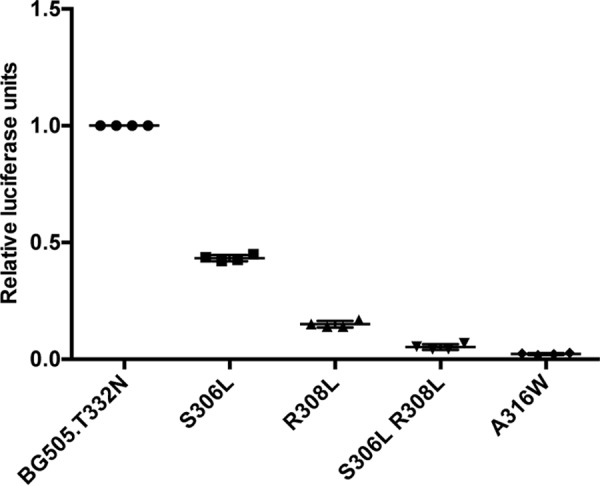
**Infectivity of BG505 pseudovirus with hydrophobic substitution in V3.** Infection of TZM-bl cells by various BG505.T332N Env-pseudovirus variants containing the S306L, R308L, and/or A316W substitutions was assessed. BG505.T332N Env-pseudoviruses were obtained via transfection of 293T HEK cells. Subsequently, an equal amount of virus input (500 pg), as determined by p24 levels in the supernatant of the 293T transfected cells, was applied to TZM-bl cells to compare the infectivity. The mean of four individual measurements within one experiment is displayed ± standard deviation.

To determine the effect of the S306L and R308L substitutions on Env function, we introduced them into the infectious BG505.T332N Env-pseudotyped virus and measured infectivity on TZM-bl cells (Fig. 2). The individual S306L and R308L substitutions decreased infectivity by 2- and 5-fold, respectively, and when combined, by 10-fold. The A316W single mutant was also poorly infectious, consistent with our earlier report (25). Thus, the introduction of hydrophobic residues at V3 positions 306, 308, and 316 compromises virus infectivity, probably by interfering with the conformational changes that expose the CCR5 co-receptor binding site or by directly affecting how V3 residues interact with the CCR5 receptor. However, we cannot exclude that effects of the V3 mutations on Env expression, Env cleavage, and Env incorporation into virions contribute to these results.

### Hydrophobic V3 residues increase trimer stability

**Figure 3.**
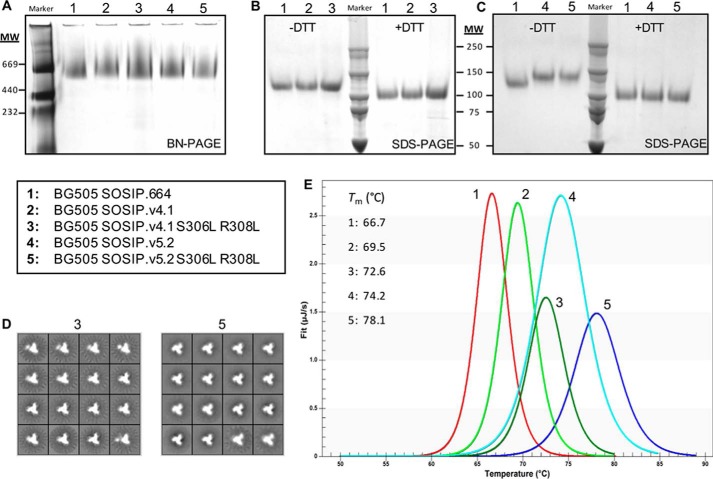
**Biochemical characterization of BG505 trimers with hydrophobic substitutions in V3.** BG505 SOSIP.v4.1 and SOSIP.v5.2 trimer variants (all containing D7324-epitope tags) were purified using PGT145-affinity chromatography and analyzed by BN-PAGE, SDS-PAGE, NS-EM, DSC, or D7324-capture ELISA. In *A–E*, the trimer variants are numbered as follows: *1*, SOSIP.664; *2*, SOSIP.v4.1; *3*, SOSIP.v4.1 S306L/R308L; *4*, SOSIP.v5.2; *5*, SOSIP.v5.2 S306L/R308L. *A*, Coomassie Blue-stained BN-PAGE gel. *B* and *C*, non-reducing (−*DTT*, *left three lanes*) and reducing (+*DTT*, *right three lanes*) SDS-PAGE gels. *D*, 2D class averages of NS-EM analyses of the SOSIP.v4.1 S306L/R308L (*3*) and SOSIP.v5.2 S306L/R308L (*5*). *E*, fitted thermal melting curves for the five trimers, analyzed using a two-state model. Data for the SOSIP.v4.1 (*2*) and SOSIP.v5.2 (*4*) trimers were adapted from Refs. 25 and 42). The unprocessed melting curves are shown in Fig. S1.

The BG505 SOSIP.v4.1 and SOSIP.v5.2 designs of stabilized Env trimer both already include the A316W substitution in V3 (25, 42). We now introduced the paired S306L and R308L substitutions into these constructs, to make the SOSIP.v4.1 S306L/R308L and SOSIP.v5.2 S306L/R308L variants. The resulting Env proteins and unmodified comparators were expressed in 293F cells, and the trimers were purified by PGT145 bNAb affinity chromatography. BN-PAGE analyses demonstrated that PGT145 affinity chromotography was a successful strategy to obtain pure Env trimers (Fig. 3*A*). The resulting trimers were each efficiently cleaved into gp120 and gp41, as judged by SDS-PAGE analyses (Fig. 3, *A–C*). In line with our previous findings, SOSIP.v5 trimers, which have an additional disulfide bond between gp120 and gp41, migrated more slowly on non-reducing SDS-PAGE gels, compared with their SOSIP.664 and SOSIP.v4 counterparts (42). The most likely explanation is a decrease in SDS uptake when a trimer is more compact. The yields of the S306L plus R308L-substituted SOSIP.v4.1 and SOSIP.v5.2 trimers, ∼1.0 and ∼0.5 mg/liter, respectively, were reduced compared with the unmodified SOSIP.v4.1 and SOSIP.v5.2 versions, ∼2.0 mg/liter in both cases (Table 2). These findings are consistent with the lower expression observed in the analyses of unpurified Env proteins (Fig. 1*E*). The S306L plus R308L-substituted trimers were predominantly (>95%) native-like when visualized by negative-stain electron microscopy (NS-EM), indicating that these V3 changes do not have global adverse effects on trimer conformation (Table 2 and Fig. 3*D*).

**Table 2:** **NS-EM and DSC data on BG505 SOSIP trimer variants** The NS-EM and DSC data are summarized from the experiments shown in Fig. 3 (*D* and *E*) and Fig. S1. The DSC data were fitted using a two-state model (Fig. 3*E*). The *T*_m_ values and NS-EM data for SOSIP.664, SOSIP.v4.1, and SOSIP.v5.2 have been reported previously and are listed here for comparison (25, 42). All the trimers contain a C-terminal D7324 tag except for SOSIP.v5.2, where a His tag was present instead (42). NL, native-like.

Construct	Yield	NS-EM (NL trimers)	DSC	
			*T*_m_	Δ*T*_m_
	*mg/liter*	%	°*C*	°*C*
BG505 SOSIP.664	∼2.0	>95	66.7	
BG505 SOSIP.v4.1	∼2.0	>95	69.5	2.7
BG505 SOSIP.v4.1 S306L R308L	∼1.0	>95	72.6	5.9
BG505 SOSIP.v5.2	∼2.0	>95	74.2	7.5
BG505 SOSIP.v5.2 S306L R308L	∼0.5	>95	78.1	11.4

We next assessed the thermostability of the SOSIP.v4.1 S306L/R308L and SOSIP.v5.2 S306L/R308L proteins and their unmodified comparators by differential scanning calorimetry (DSC). The midpoint of thermal denaturation (*T*_m_) of 72.6 °C for the SOSIP.v4.1 S306L/R308L trimer was higher than the value of 69.5 °C for SOSIP.v4.1, and likewise for SOSIP.v5.2 S306L/R308L compared with SOSIP.v5.2 (78.1 °C *versus* 74.2 °C; Fig. 3*E* and Fig. S1). Thus, the S306L and R308L substitutions increased trimer thermostability by 3–4 °C, which is consistent with our earlier finding for the A316W change (43). The explanation may be that the two introduced leucine residues strengthen the hydrophobic packing of the V3 region within the pocket underneath the V1V2 domain (Table 1 and Fig. 3*E*). The stabilization effect of the two substitutions described here and the previously described substitutions A316W and E64K, and the 73C–561C disulfide bond are additive. When all substitutions are considered in the BG505 SOSIP.v5.2 S306L/R308L trimer, its stability is increased by 11.4 °C (from BG505 SOSIP.664 to BG505 SOSIP.v5.2 S306L/R308L; Table 1 and Fig. 3*E*).

### Hydrophobic V3 residues decrease binding of V3 mAbs to trimers

**Figure 4.**
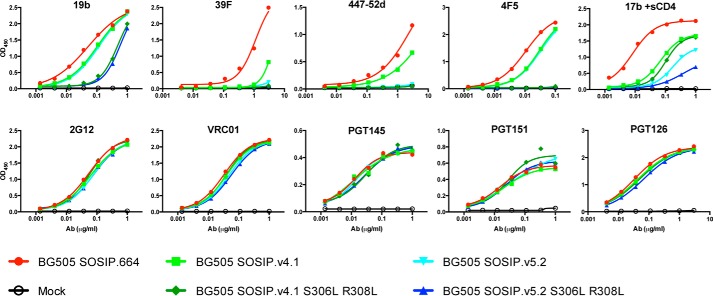
**Antigenicity of BG505 trimers with hydrophobic substitutions in V3.** mAb binding to the various PGT145-purified BG505 trimer variants was measured with a D7324 capture ELISA. The binding to BG505 trimer variants of several neutralizing and non-neutralizing mAbs, including four V3 targeting non-neutralizing mAbs, was assessed.

The antigenicity of the same five PGT145-purified trimers was studied by ELISA, to further assess whether the S306L and R308L substitutions had decreased the presentation of the V3 epitopes for mAbs 39F, 4F5, 447–52D, and 19b. All four mAbs bound efficiently to the SOSIP.664 trimers, consistent with previous observations that under the conditions of an ELISA, but not SPR, ITC, or NS-EM, the V3 mAb epitopes are accessible (Fig. 4) (13, 25). Compared with SOSIP.664, the four V3 mAbs bound 2–10-fold less efficiently to the SOSIP.v4.1 trimer, whereas 39f and 447–52D did not bind detectably to SOSIP.v5.2 (Fig. 4), which is consistent with observations made elsewhere (25, 42). When the S306L plus R308L substitutions were present in the SOSIP.v4.1 and SOSIP.v5.2 constructs, there was no detectable binding of 39f, 4F5, and 447–52D, and 19b binding was further reduced compared with the unmodified trimers (Fig. 4).

As noted above, the leucine substitutions might impair V3 non-NAb binding either indirectly by limiting the overall accessibility of the V3 domain, directly by disrupting the epitope(s), or by a combination of these effects. The 17b non-NAb interacts with a CD4-induced epitope associated with the CCR5-binding site (45, 46). We observed that the ability of soluble CD4 to induce the exposure of the 17b epitope was strongly impaired for trimers containing the S306L plus R308L substitutions (Fig. 4). The implication is that the enhanced hydrophobic packing of the V3 region interferes with CD4-induced conformational changes in the trimer. By extension, the same restrictions to conformational flexibility could contribute to the reduced antigenicity of the V3 non-NAb epitopes.

Converse to the reduction in V3 antigenicity, the S306L plus R308L paired substitutions had no effect on the trimer reactivity of bNAbs targeting other epitopes (2G12, VRC01, PGT145, PGT151, and PGT126), including those that recognize quaternary epitopes that are highly sensitive to the overall conformation of the trimer (Fig. 4). Thus, consistent with the NS-EM images (Fig. 3*D*), the leucine substitutions in V3 do not have a globally adverse effect on trimer conformation.

### Reduced V3 non-NAb induction in trimer-immunized rabbits

**Figure 5.**
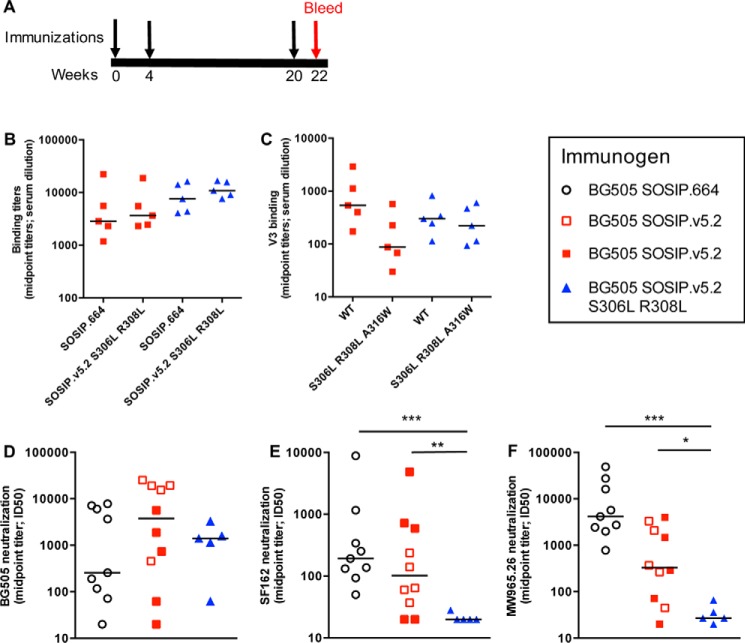
**Immunogenicity of BG505 SOSIP.v5.2 S306L/R308L trimers in rabbits.** *A*, immunization schedule. The rabbits were immunized with 22 μg of the BG505 SOSIP.v5.2 S306L/R308L and comparator proteins at weeks 0, 4, and 20, and Ab responses were measured at week 22. *B*, midpoint anti-trimer binding titers were determined in a D7324-capture ELISA based on the BG505 SOSIP.664 and SOSIP.v5.2 S306L/R308L trimers. *C*, midpoint binding titers were determined in a peptide ELISA using a cyclized BG505 V3 peptide (WT) and a variant containing the S306L, R308L, and A316W changes that are present in the SOSIP.v5.2 S306L/R308L trimer. *D–F*, midpoint neutralizing titers against the autologous tier 2 virus BG505.T332N (*D*) and the heterologous tier 1A viruses SF162 and MW965.26 (*E* and *F*) were determined using the TZM-bl assay. To assist in the overall interpretation of the immunization experiment, we included additional sera from other rabbit studies (see “Experimental procedures”). Specifically, these were nine sera from BG505 SOSIP.664 recipients (*open black circles*) and five sera from BG505 SOSIP.v5.2 recipients (*open red squares*). Mann–Whitney U tests were used to determine whether differences between groups were statistically significant (*, *p* < 0.05; **, *p* < 0.01; ***, *p* < 0.001).

The *in vitro* studies demonstrate that the paired leucine substitutions in V3 increase the thermostability of BG505 SOSIP trimers and reduce their antigenicity for V3 non-NAbs without compromising the overall conformation of the trimer. Accordingly, we assessed whether the modifications affected the immunogenicity of trimers *in vivo* using rabbits. Specifically, we immunized rabbits with PGT145-purified BG505 SOSIP.v5.2 or SOSIP.v5.2 S306L/R308L trimers at weeks 0, 4 and 20, and compared trimer-binding and virus-neutralizing Ab responses at week 22 (Fig. 5*A*).

We first determined binding Ab titers to the BG505 SOSIP.v5.2 S306L/R308L and SOSIP.664 trimers (Fig. 5*B*). Sera from the SOSIP.v5.2 S306L/R308L-immunized rabbits bound comparably to the sequence-modified and parental trimers, indicating that the induced Abs predominantly recognized epitopes shared between these two trimers. This finding was also true of sera from the SOSIP.v5.2-immunized rabbits (Fig. 5*B*).

To gain insights into the V3 Ab response, we measured Ab titers to the wildtype BG505 V3 peptide and a variant containing the S306L/R308L/A316W sequence changes present in the SOSIP.v5.2 S306L/R308L trimer (Fig. 5*C*). The sera from the SOSIP.v5.2-immunized bound less efficiently (6-fold lower median titer) to the triple mutant peptide compared with the wildtype peptide, implying that residues Ser^306^, Arg^308^, and Ala^316^ contribute to the overall immunogenicity of the V3 region of the SOSIP.v5.2 trimer. Sera from the SOSIP.v5.2 S306L/R308L recipients bound comparably to the two V3 peptide variants. These sera bound marginally less well (2-fold reduction in median titer) to the wildtype peptide, compared with the sera from the SOSIP.v5.2-immunized rabbits. Overall, we conclude from the V3-peptide serology studies that the V3 domain on the SOSIP.v5.2 S306L/R308L trimer was a little less immunogenic than on the SOSIP.v5.2 version and that the introduced leucine residues did not create highly immunogenic V3 neo-epitopes (Fig. 5*C*).

Next, we measured neutralization of the autologous tier 2 virus BG505.T332N virus. In all of the NAb assay analyses, we also included sera from rabbits that had been immunized with either BG505 SOSIP.664 (9 animals, *open circles*) or SOSIP.v5.2 (5 animals, *open squares*) trimers in earlier experiments using an identical immunization schedule (25, 31, 42). We have reported elsewhere that the magnitude of the autologous NAb response to BG505 SOSIP trimers is highly variable among rabbits, which limits the statistical power of intergroup comparisons when the group sizes are small (47). This was also the case in the present study (Fig. 5*D*). There was a generally strong autologous NAb response in the SOSIP.v5.2 and SOSIP.v5.2 S306L/R308L immunized rabbits, with median titers of 3761 and 1415, respectively. The median titer for the historic control group of SOSIP.664 immunized rabbits was 257 (Fig. 5*D* and Table 3) (25, 31, 47). The differences between the median titers were not statistically significant (*p* = 0.28 and *p* = 0.84). We conclude that the introduction of hydrophobic residues in V3 does not compromise the autologous NAb response to SOSIP trimers. This finding is consistent with observations that the autologous NAb response induced by BG505 SOSIP trimers does not target V3 but is directed elsewhere, for example against a hole in the glycan shield at positions 241 and/or 289 (25, 31, 47, 48).

**Table 3:**
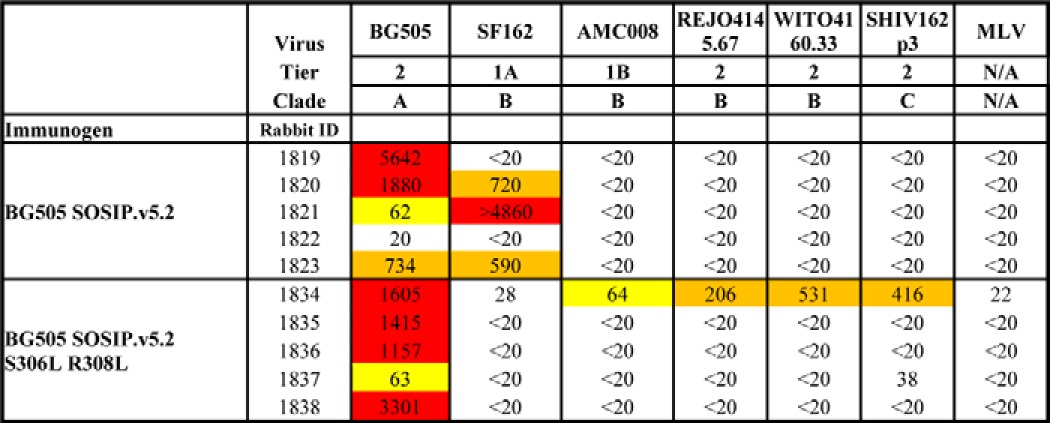
**Midpoint neutralization titers for sera from rabbits immunized with BG505 SOSIP.v5.2 or SOSIP.v5.2 S306L/R308L trimers, tested against a panel of Env-pseudotyped viruses** The neutralization data were derived at the Academic Medical Center. The ID_50_ values for the week 22 sera are recorded, *i.e.* the serum dilution at which infectivity was inhibited by 50%. The boxes are colored as follows: white, ID_50_ < 40 = no neutralization; yellow, ID_50_ 40–100 = weak neutralization; orange, ID_50_ 101–1000 = moderate neutralization; red, ID_50_ > 1000 = strong neutralization. MLV = murine leukemia virus (negative control).

Tier 1A viruses that are highly neutralization sensitive are particularly vulnerable to V3 Abs that lack the ability to neutralize tier 2 viruses such as, but not limited to, BG505.T332N (14). We have previously shown that BG505 SOSIP.664 trimers induce strong, V3-dependent NAb responses against the clade B tier 1A virus SF162 (25, 31). Compared with the historic control data for SOSIP.664-immunized rabbits, sera from the SOSIP.v5.2 recipients neutralized SF162 at an ∼2-fold lower titer (median titers of 192 *versus* 102; Fig. 5*E*). In contrast, four of the five sera from the SOSIP.v5.2 S306L/R308L-immunized rabbits lacked any ability to neutralize the SF162 virus, and only a minimal response was found for the fifth serum (ID_50_ of 27; Fig. 5*E* and Table 3). Overall, there was a 10-fold reduction in median SF162 ID_50_ for the SOSIP.v5.2 S306L/R308L group compared with SOSIP.664 (*p* = 0.001) and a 5-fold reduction compared with SOSIP.v5.2 (*p* = 0.02). Similar results were obtained using the clade C tier 1A virus MW965.26 (Fig. 5*F* and Table 3). Thus, the median ID_50_ against this virus was 4166 for the SOSIP.664 historic control group, 328 for SOSIP.v5.2, but only 27 for SOSIP.v5.2 S306L/R308L. Compared with SOSIP.664 and SOSIP.v5.2, a highly significant reduction in MW965.26 neutralization was seen for SOSIP.v5.2 S306L/R308L immunized rabbits of 154- and 12-fold respectively (*p* = 0.01 for SOSIP.v5.2 S306L/R308L *versus* SOSIP.v5.2 and *p* = 0.001 for SOSIP.v5.2 S306L/R308L *versus* SOSIP.664, respectively).

None of the sera consistently neutralized any of the heterologous tier 2 viruses tested, except for animal 1834, which received BG505 SOSIP.v5.2 S306L/R308L (Tables 3 and 4). The serum from this animal neutralized the REJO4145.67, WITO4160.33, and SHIV162p3 viruses with ID_50_ values of 206, 531, and 416 (Table 3). We do not know whether this unusually strong cross-neutralizing response is attributable to chance or whether the introduction of the S306L and R308L substitutions played a role. A larger study will be required to probe this question further. In summary, the tier 1A NAb responses, but not the autologous tier 2 NAb responses, in BG505 SOSIP trimer-immunized rabbits were reduced by the introduction of sequence changes in V3 that were designed to reduce the exposure and/or immunogenicity of the V3 region.

**Table 4:**
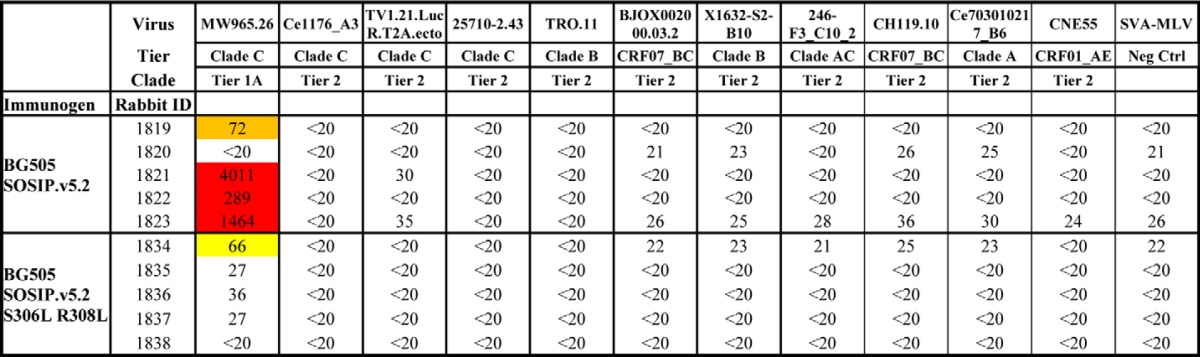
**Midpoint neutralization titers for sera from rabbits immunized with BG505 SOSIP.v5.2 or SOSIP.v5.2 S306L/R308L trimers, tested against a panel of Env-pseudotyped viruses** The neutralization data were derived at the Duke University Medical Center. The ID_50_ values for the week 22 sera are recorded, *i.e.* the serum dilution at which infectivity was inhibited by 50%. The boxes are colored as follows: white, ID_50_ < 40 = no neutralization; yellow, ID_50_ 40–100 = weak neutralization; orange, ID_50_ 101–1000 = moderate neutralization; red, ID_50_ > 1000 = strong neutralization. SVA-MLV = murine leukemia virus (negative control).

## Discussion

The various designs and genotypes of native-like SOSIP trimers now serve as a platform for the eventual induction of bNAbs, which is a challenging task that will require manipulating the immunogen design so as to reproduce consistently how bNAbs are sometimes elicited during HIV-1 infection (49, 50). One substantial unknown at the interface of immunogen design and immunology is whether, and to what extent, “off-target” epitopes for non-NAbs interfere with the more desirable Ab responses that are, or may be, on the pathway toward bNAbs. From the perspective of SOSIP trimer design, immunogenicity studies in rabbits and macaques have shown that the BG505 and other genotypes of the SOSIP.644 construct induce substantial titers of Abs against the V3 region that neutralize only tier 1 viruses, as well as the more desirable autologous tier 2 NAbs against more complex epitopes that appear to involve interaction with residues that are exposed via isolate-specific holes in the glycan shield (14, 25, 47, 48). These two categories of Ab response are non-correlated. We have also shown that the immunogenicity of the V3 region in rabbits can be reduced by the introduction of an A316W substitution that helps to lock the V3 region within the body of the trimer and thereby reduces its surface exposure (25). Thus, the resulting BG505 SOSIP.v4.1 trimer induced anti-V3 Ab titers and NAb titers against tier 1A viruses, which are dominated by V3 Abs that were reduced by ∼10-fold, respectively, compared with SOSIP.664. This decrease in V3 immunogenicity was not accompanied by a substantial change in the autologous tier 2 NAb response (25). The question remains, however, whether a greater reduction in the induction of off-target responses could have a beneficial effect, and whether it would facilitate the eventual development of bNAbs.

Here, we describe the introduction of further, structure-guided modifications to the V3 region of SOSIP.v4.1 and SOSIP.v5.2 trimers, specifically the introduction of two hydrophobic residues, S306L and R308L, within V3. The structure of the prefusion conformation of the trimer shows that V3 is sequestered within a hydrophobic pocket below the V1V2 domain. We sought to further stabilize the interdomain interactions in that section of the trimer, to reduce the propensity of V3 to adopt a more exposed position in which it could be recognized by the humoral immune system. This goal was successfully accomplished; the S306L and R308L substitutions reduced V3 mAb binding to the resulting trimers and reduced CD4-induced conformational changes. The modified trimers were also more thermostable. Single substitutions only partially reduced V3 exposure and the introduction of three hydrophobic residues via the S306L, R308L, and A316W changes was necessary for the full effect. *In silico* analyses showed that all three of the introduced residues are in close contact (<5 Å) with neighboring non-polar amino acids, suggesting that the hydrophobic interactions are the driving force for V3 stabilization (Table 1 and Fig. 1*D*). The same *in silico* studies showed the importance of limiting clashes between introduced hydrophobic residues and their neighbors. For example, an L125F substitution in the V1V2 stem created a clash with an aromatic residue in V3 (Phe^317^) and resulted in an increase in V3 exposure on the Env spike of the JRFL virion (44).

When rabbits were immunized with the BG505 SOSIP.v5.2 S306L/R308L trimer, the induction of V3-targeting tier 1A SF162 NAbs was reduced by 10- and 5-fold compared with SOSIP.664 or SOSIP.v5.2, respectively. Despite the almost undetectable levels of tier 1A NAbs, the autologous tier 2 NAb responses were comparable in all three of the immunization groups. Thus, at least in this type of immunization strategy, we could find no evidence for immunodominant non-NAb V3 epitopes interfering with the induction of tier 2 NAbs. Again, we note that these two categories of NAb responses to SOSIP trimers are generated independently of each other (14, 25).

We have considered whether the leucine substitutions at positions 306 and 308 might also directly affect the immunogenicity of the V3 region by destroying existing epitopes and/or by creating neo-epitopes. Human mAbs 447–52D and 2219 that have some capacity to neutralize tier 2 viruses bind the GPGR motif at the V3 crown but also interact with residues 306 and 308 (51–53). The epitopes for many other mAbs with lesser neutralization capacity also involve charged residues flanking the V3 crown (54). However, this observation is not universally true; the 19b non-NAb epitope does not depend on residues 306 and 308 (Table 5). When V3 residues 306–311 were replaced by serine in the gp120 monomer context, the immunogenicity of this region was reduced in mice (55). Thus, the S306L and R308L substitutions clearly do affect an immunogenic region of V3 (Table 5). It is therefore likely that the lower tier 1 NAb responses to the modified trimers reflect a combination of reducing V3 exposure and diminishing the response to some epitopes if and when V3 does become accessible. We cannot assess the relative contributions of these two mechanisms. However, we could find no evidence that the leucine substitutions created immunodominant neo-epitopes (Fig. 5*C*).

**Table 5:** **Interaction of anti-V3 mAbs with residues 306, 308, and 316** The recorded dependencies of V3 mAbs 447–52D, 19b, and 39f on residues 306, 308, and 316 are derived from published reports (51, 54, 89). Although the dependencies of 19b and 39f are based on neutralization experiments, the dependency of 447–52D is based on the interactions observed in a crystal structure with a V3 peptide. A plus sign indicates the residue directly contributes to the MAb epitope, and a minus sign indicates that it does not. There is no published information on the other V3 mAbs used in this study, 14e and 4F5.

	Ser/Arg^306^	Arg/His^308^	Ala^316^
447-52D	+	+	−
19b	−	−	−
39f	−	+	−

The strategy of reducing the immunogenicity of V3 non-NAb epitopes is based on the hypothesis that the subdominance of (b)NAb epitopes would be alleviated (56, 57). However, do off-target non-NAb responses interfere with (b)NAb development or are they merely irrelevant? Non-NAb interference has never been proven in HIV-1 Env immunization studies, and we could find no evidence for it in this study. However, there remain several arguments, some supported by experimental evidence, in favor of reducing non-NAb epitope immunodominance. For example, non-NAbs have been proposed to interfere with the induction of bNAbs against several pathogens, such as influenza and malaria (58–63). In the HIV-1 vaccine context, germline precursors of non-NAbs, *i.e.* the BCR on naive B-cells, are abundant in the human Ab repertoire, are easily activated by various Env immunogens, and need relatively few somatic hypermutation events to acquire high affinity. In contrast, the germline precursors of bNAbs are rare, are difficult to activate by Env immunogens, and require extensive SHM to become high affinity bNAbs (56). *In vitro* B-cell activation studies suggest that because germline non-NAb precursors have a higher affinity for Env proteins, they have a selection advantage in the germinal center over the germline bNAb precursors (57). In this germinal center context, the selection of higher-affinity B cell clones after Env protein immunization hinders the activation and affinity maturation of low-affinity germline bNAbs that compete for the same resources (56, 57, 64). By extrapolation, there is a good argument for preventing the activation of high-affinity non-NAb precursors and thereby promoting the activation of their lower affinity bNAb counterparts (56, 64, 65). Thus, it may be beneficial to reduce the immunogenicity of V3 and other non-NAb epitopes when designing immunogens to initiate and mature bNAb lineages (39, 66–68). The strategies that we have described should therefore be tested in that context. Whether this approach or other approaches to diminish V3 immunogenicity are superior in this context remains to be tested experimentally (34, 39, 41).

In summary, we have used structure-based design to generate next generation BG505 SOSIP trimers on which the immunogenicity of V3 non-NAb epitopes is strongly reduced. This outcome was achieved via the S306L and R308L hydrophobic substitutions, which complement the previously described A316W change. The general method of suppressing V3 immunogenicity may be useful for various strategies aimed at inducing bNAbs, such as germline targeting (65, 68–72), patient-based lineage immunogens (73–78) and particulate immunogens (66, 79–82).

## Experimental procedures

### Construct design

The BG505 SOSIP.664 construct has been described elsewhere (14). SOSIP.664-D7324 trimers contain a C-terminal D7324 epitope-tag sequence and were constructed by adding the amino acid sequence GSAPTKAKRRVVQREKR after residue 664 of gp41_ECTO_ (13). The following sequence changes to the SOSIP.664 introduce additional stability: E64K plus A316W to generate SOSIP.v4.1 (25) and E64K plus A316W and the A73C-A561C intersubunit disulfide bond to make SOSIP.v5.2 (42). All mutant constructs were made using the QuikChange site-directed mutagenesis system (Agilent, Stratagene) and verified by sequencing. D7324-tagged versions of these various trimers were used for all the *in vitro* experiments, but untagged trimers were tested as immunogens in rabbits.

### Env protein expression

The various SOSIP Env constructs were expressed by polyethyleneimine-mediated transient transfection (together with the *furin* gene) of adherent human embryonic kidney (HEK) 293T cells or of HEK 293F cells adapted for serum-free suspension cultures, as previously described (13, 25). For small-scale trimer expression, 293T cells were used, and for larger-scale trimer production, 293F cells were used. All experiments with purified trimers utilized 293F cell-expressed proteins.

### Trimer purification

For screening purposes, unpurified 293T-expressed trimers were analyzed directly using the culture supernatants obtained 6 days post-transfection. For extensive analysis of trimers with reduced V3 reactivity, Env trimers were purified from 293F transfection supernatants by affinity chromatography using a PGT145 column, essentially as described (13, 25, 83). Protein concentrations were determined using UV280 absorbance and theoretical extinction coefficients via ExPASy (ProtParam tool). All experiments were performed with D7324 tagged trimers, except for the immunization studies, for which untagged trimers were used.

### SDS-PAGE and BN-PAGE

Env proteins were analyzed using SDS-PAGE and BN-PAGE, followed by Western blotting or Coomassie Blue dye staining as previously described (25, 84, 85).

### Negative-stain EM

PGT145-purified BG505 trimers were analyzed by negative-stain EM as previously described (17, 25, 83).

### Differential scanning calorimetry

Thermal denaturation of PGT145-purified BG505 trimers was studied using a MicroCal VP-Capillary DSC calorimeter (Malvern Instruments) or a nano-DSC calorimeter (TA Instruments) as described previously (13, 25). The data were fitted using both two-state and non–two-state models, because the asymmetry of some of the peaks suggested that unfolding intermediates were present. We report the *T*_m_ values derived from the two-state model in Table 2 and Fig. 3*E*, whereas the multiple *T*_m_ values based on the non–two-state models are shown in Fig. S1.

### Rabbit immunization

Rabbits were immunized at weeks 0, 4, and 20 at Covance as described previously, using 22 μg of PGT145-purified trimers formulated in ISCOMATRIX^TM^ adjuvant (25). Binding Ab and NAb responses were assessed in sera from blood samples drawn at week 22, as described below. Rabbit IDs were 1274–1277, 1569–1573 (14, 25), 1589–1593 (42), 1819–1823, and 1834–1838.

### D7324-capture ELISA

The methods to perform sandwich ELISAs using D7324-tagged BG505 SOSIP.664 trimers have been described elsewhere (13, 86). V3 mAb 4F5 was isolated from a patient participating in the Amsterdam Cohort Studies on HIV/AIDS (patient H18877) (87). The other mAbs used here have been described elsewhere (75, 88, 90, 91). To study binding of 17b to BG505 trimer variant in the presence of CD4 binding, 1 μg/ml soluble CD4 (sCD4) was added during the primary Ab incubation step with serially diluted 17b. To quantify Ab responses in immunized rabbits, sera (from week 22) were serially diluted in 3-fold steps from a 1:100 start point, using 40% sheep serum (Biotrading), 2% milk powder in TBS as the buffer. We used BG505 SOSIP.D7324 and SOSIP.v5.2 S306L/R308L trimers (1 μg/ml) to quantify trimer binding titers in rabbit sera and BG505 V3 peptide to quantify anti-V3 titers (see below).

### V3 peptide ELISA

To determine V3 Ab responses in BG505-immunized rabbits, 96-well MaxiSorp plates were coated overnight at 4 °C with 2 μg/ml of a cyclized V3-peptide in PBS. The V3 peptide was based on the sequence of the BG505 SOSIP immunogen, either unmodified (CTRPNNNTRKSIRIGPQAFYATGDIIGDIRQAHC) or including the S306L, R308L, and A316W changes highlighted in bold (CTRPNNNTRK**L**I**L**IGPQ**W**FYATGDIIGDIRQAHC). The V3 peptides were cyclized by a disulfide bond between residues 1 (residue 296 in HXB2 gp160 numbering) and 35 (residue 331). The plates were then washed with PBS supplemented with 0.1% Tween 20 and blocked for 1.5 h at room temperature with PBS supplemented with 0.05% Tween 20, 3.3% FBS, and 2% BSA (PBS-TFB). After washing with PBS, 0.1% Tween, rabbit serum dilutions were added in PBS-TFB. The subsequent steps in the assay were as described for the D7324 trimer-capture ELISA previously (13, 25).

### Single-round infection assay and neutralization assay

The TZM-bl reporter cell line, which stably expresses high levels of CD4 and the co-receptors CCR5 and CXCR4 and contains the luciferase and β-galactosidase genes under the control of the HIV-1 long-terminal-repeat promoter, was obtained through the National Institutes of Health AIDS Research and Reference Reagent Program, Division of AIDS, NIAID, National Institutes of Health (John C. Kappes, Xiaoyun Wu, and Tranzyme Inc., Durham, NC). TZM-bl cell neutralization assays using Env-pseudotyped viruses were performed at two different sites: Duke University Medical Center, Durham, NC (for methodology see Ref. 92) and Academic Medical Center, Amsterdam (for methodology see Refs. 14 and 25). The assays at Duke University Medical Center were performed essentially as described in protocols at: http://www.hiv.lanl.gov/content/nab-reference-strains/html/home.htm. The small modifications in the assays performed at the AMC compared with the Duke University Medical Center were also described previously (14, 25).

## Author contributions

S. W. d. T., D. C. M., I. A. W., J. P. M., A. B. W., and R. W. S. conceptualization; S. W. d. T. and A. T. d. l. P. data curation; S. W. d. T., A. T. d. l. P., A. V., I. A. W., J. P. M., A. B. W., and R. W. S. formal analysis; S. W. d. T., A. T. d. l. P., and J. P. M. validation; S. W. d. T., A. T. d. l. P., A. V., E. S., K. S., J. A. B., P. v. d. W., A. S., E. E. S., C. C. L., and R. W. S. investigation; S. W. d. T. visualization; S. W. d. T., A. V., E. S., K. S., J. A. B., P. v. d. W., A. S., E. E. S., and C. C. L. methodology; S. W. d. T., J. P. M., and R. W. S. writing-original draft; S. W. d. T. and C. C. L. project administration; S. W. d. T., A. T. d. l. P., J. A. B., M. J. v. G., I. A. W., J. P. M., A. B. W., and R. W. S. writing-review and editing; M. J. v. G., D. C. M., A. B. W., and R. W. S. resources; A. B. W. and R. W. S. supervision; R. W. S. funding acquisition.

## Supplementary Material

Supporting Information

## Footnotes

4 The abbreviations used are:

NAb: neutralizing antibody
bNAb: broadly neutralizing antibody
Env: envelope glycoprotein
DSC: differential scanning calorimetry
NS: negative-stain
BN: Blue Native.
