# Supplementary material for: Stabilization of the gp120 V3 loop through hydrophobic interactions reduces the immunodominant V3-directed non-neutralizing response to HIV-1 envelope trimers
Source: J Biol Chem. 2017 Dec 7;293(5):1688–701. doi: 10.1074/jbc.RA117.000709 (PMC5798299; doi:10.1074/jbc.RA117.000709)
Supplement: Supporting Information [file supp_293_5_1688__index.html]

Stabilization of the gp120 V3 loop through hydrophobic interactions reduces the immunodominant V3-directed non-neutralizing response to HIV-1 envelope trimers — V3 exposure on HIV-1 Env trimers — Stabilization of the gp120 V3 loop through hydrophobic interactions reduces the immunodominant V3-directed non-neutralizing response to HIV-1 envelope trimers — V3 exposure on HIV-1 Env trimers — Supporting Information 

# Stabilization of the gp120 V3 loop through hydrophobic interactions reduces the immunodominant V3-directed non-neutralizing response to HIV-1 envelope trimers

## Supporting Information

- S1 - Figure S1
