# Supplementary figures and images for: Stabilization of the gp120 V3 loop through hydrophobic interactions reduces the immunodominant V3-directed non-neutralizing response to HIV-1 envelope trimers
Source: J Biol Chem. 2017 Dec 7;293(5):1688–701. doi: 10.1074/jbc.RA117.000709 (PMC5798299; doi:10.1074/jbc.RA117.000709)

Figure S1

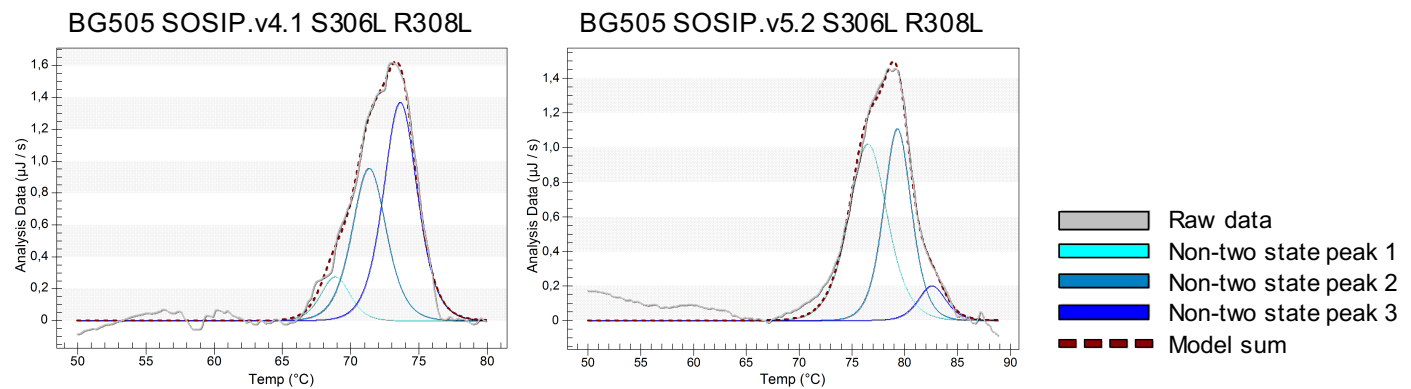

Supplement: Supporting Information [file supp_RA117.000709_133553_1_figure_32154_p0j27n.pdf]
